# Simplifying the B Complex: How Vitamins B6 and B9 Modulate One Carbon Metabolism in Cancer and Beyond

**DOI:** 10.3390/metabo12100961

**Published:** 2022-10-11

**Authors:** Carolina N. Franco, Laurence J. Seabrook, Steven T. Nguyen, Jack T. Leonard, Lauren V. Albrecht

**Affiliations:** 1 Department of Pharmaceutical Sciences, University of California, Irvine, CA 92697, USA; 2 Department of Developmental and Cell Biology, University of California, Irvine, CA 92697, USA; 3 Department of Chemistry, University of California, Irvine, CA 92697, USA

**Keywords:** folate, pyridoxine, methionine, one carbon metabolism, cancer, methylation, B6, B9, post-translational modification, CEST-MRI, aptamer, metabolic probe, fluorescent sensors

## Abstract

Vitamin B micronutrients are essential regulators of one carbon metabolism that ensures human health. Vitamin B9, or folate, lies at the heart of the folate cycle and converges with the methionine cycle to complete the one carbon pathway. Additionally, vitamin B6 contributes by orchestrating the flux of one carbon cycling. Dysregulation of vitamin B contributes to altered biochemical signaling that manifests in a spectrum of human diseases. This review presents an analysis of the past, present, and future work, highlighting the interplay between folate and vitamin B6 in one carbon metabolism. Emerging insights include advances in metabolomic-based mass spectrometry and the use of live-cell metabolic labeling. Cancer is used as a focal point to dissect vitamin crosstalk and highlight new insights into the roles of folate and vitamin B6 in metabolic control. This collection of vitamin-based research detailing the trends of one carbon metabolism in human disease exemplifies how the future of personalized medicine could unfold using this new base of knowledge and ultimately provide next-generation therapeutics.

## 1. Introduction

**Figure 1:**
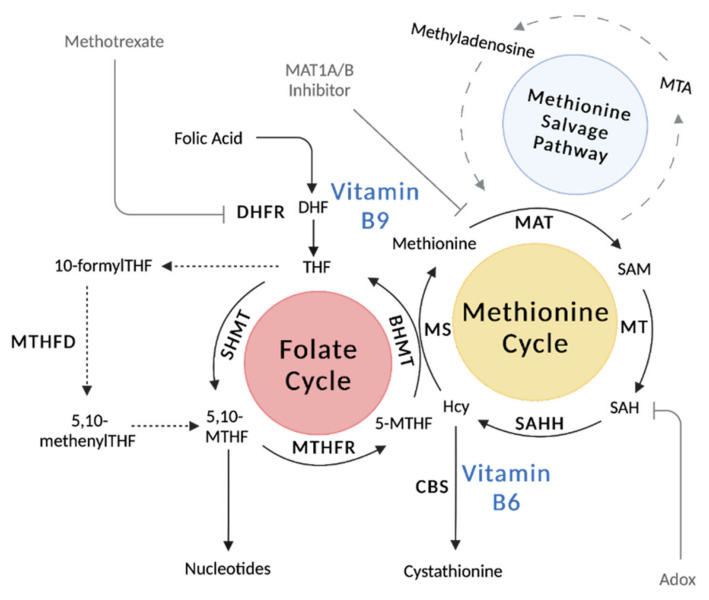
One carbon metabolism is a collection of cyclical metabolic pathways that orchestrates myriad metabolic processes. The one carbon pathway is comprised of the folate cycle and the methionine cycle. Folate enters the pathway through a two-step reaction that generates tetrahydrofolate (THF) with the dihydrofolate reductase enzyme (DHFR). Within the methionine cycle, dietary methionine is catabolized by methionine adenosyltransferase 2A or 1A (MAT2A/MAT1A) to produce the universal methyl donor S-adenosylmethionine (SAM). Upon substrate methylation, SAM is converted to S-adenosylhomocysteine (SAH) and then converted by adenosylhomocysteinase (AHCY) to homocysteine (Hcy). The methionine cycle is completed by the conversion of Hcy back to methionine by methionine synthase (MS). Alternatively, Hcy can fed into the transsulfuration pathway for glutathione synthesis and redox metabolism using vitamin B6-dependent enzymes. The methionine salvage pathway also sparks off the methionine cycle to produce the by-product methylthioadenosine (MTA) from methionine for polyamine biosynthesis. Figure made with BioRender.

Vitamins dynamically orchestrate cellular metabolic pathways to regulate human physiology. One carbon metabolism is a vital pathway across living organisms that relies on the levels of several vitamins from the B family. Vitamin B micronutrients are water-soluble compounds obtained from diet. The era of vitamin discovery occurred in the early 20th century and were aptly named as ‘vital amines’ or ‘vitamines’ due to the common amine in the chemical structure. While researchers had begun to understand the importance of this new class of nutrients, Casimir Funk is attributed with the discovery and naming of ‘vitamins’ in 1912; a flurry of other vitamins were discovered shortly after, including the B vitamins [1]. Researchers realized that deficiencies in this new class of molecules were the cause of several diseases, including scurvy, anemia, and rickets. Over the past century, the pioneering work of many researchers laid the groundwork of vitamin biology. Many excellent review articles cover the folate cycle and the one carbon pathway [2,3]. This review focuses on the central roles of vitamin B6 (pyridoxine) and B9 (folate), essential elements of the one carbon pathway that contribute to functional activity in health and dysregulation in disease (Figure 1). Folate enters the one carbon pathway through active forms of tetrahydrofolate (THF) that carry methyl-groups throughout the folate cycle. Pyridoxine is similarly obtained through diet and once converted to the most metabolically active form, pyridoxal 5′-phosphate (PLP), is a crucial cofactor during one carbon metabolism. This review highlights the recent advances that have revolutionized our understanding of B6 and B9 vitamins and how these lines of research offer novel insight to improve treatments in disease.

## 2. Vitamin B9: Folate

### 2.1. Dietary and Active Forms 

Vitamin B9, or folate, can be obtained directly from diet via leafy greens, seeds, and fruit juice as 5-methyltetrahydrofolate (5-MTHF) and in fortified grains or supplements as folic acid. Originally discovered in 1931, Dr. Lucy Wills found that this micronutrient was able to treat anemia during pregnancy [4,5]. Since then, the mechanistic underpinnings of vitamin B9 have been described in embryonic development [6], redox homeostasis [7], immunology [8], and cancer [9]. Dietary B9 recommendation for adults in the United States is set to 400 μg/day (CDC.org). Circulating B9 levels in adults range from 2 to 20 ng/mL [10] where the highest concentrations are the most active B9 vitamer 5-methyltetrahydrofolate (5-MTHF) at 5 μM [11]. Folate can be assessed in patients through various approaches, such as by measuring the levels of circulating homocysteine (Hcy) or by urinary formiminoglutamate (FIGLU) excretion. Additionally, folate can be directly measured in red blood cells and through deoxyuridine suppression tests [10].

### 2.2. Metabolic Pathways and Key Enzymes of the Folate Cycle 

**Figure 2:**
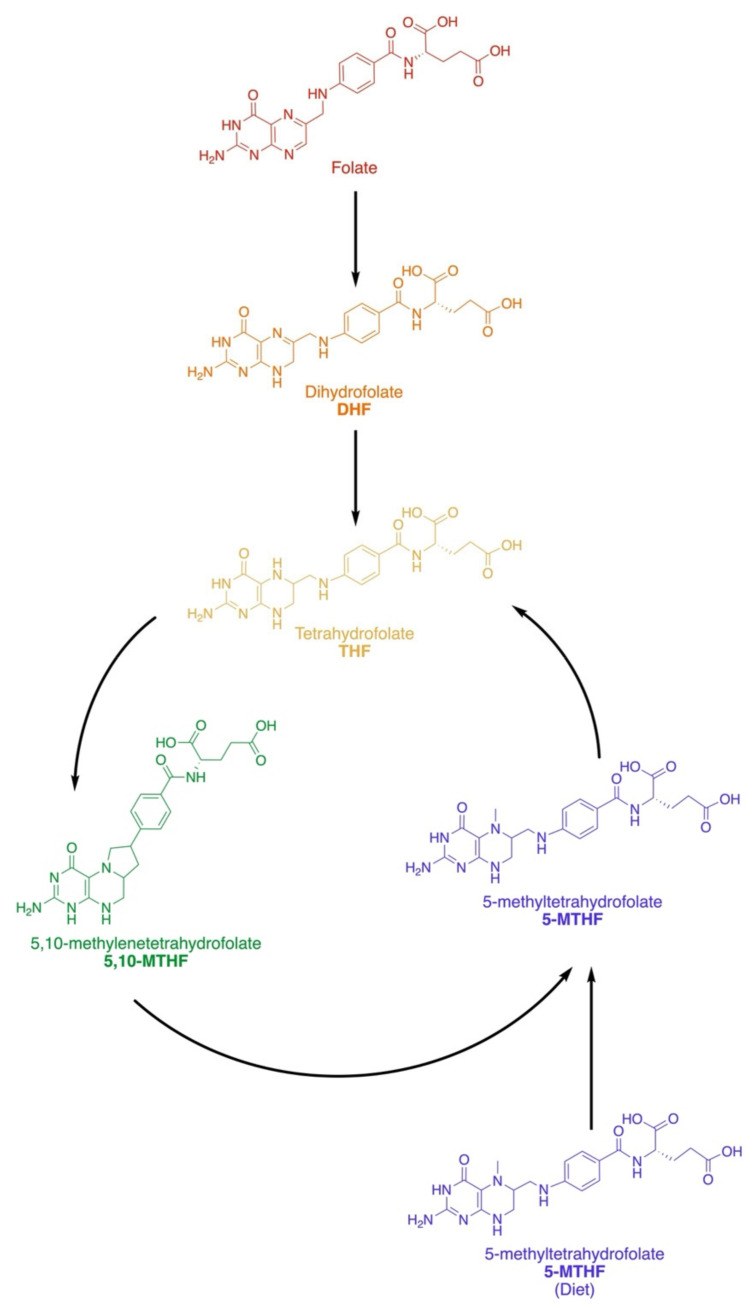
Vitamin B9 and the Folate Family. Folate or folic acid is obtained from diet and must be converted to tetrahydrofolate (THF) before it can enter the folate cycle. Dietary 5-methyl-THF can also become incorporated. Folate is converted first to dihydrofolate (DHF) before becoming THF, the universal methyl-acceptor of the one carbon pathway. Methylation can occur on the 5 or 10 N groups of THF in 5,10-methylene-THF and 5-methyl-THF.

The folate family contains a 2-amino-4-hydroxy-pteridine ring and a p-aminobenzoyl moiety linked by a methylene (CH_2_) group, which are then linked through an amide bond to the α-amino group of a monoglutamate or poly-γ-glutamate (Figure 2). Methyl groups occupy the N5 and N10 positions across vitamers [12]. Tetrahydrofolate (THF) is generated from dietary folic acid and operates as the universal one carbon acceptor [7], where the oxidation states of methyl-units include methanol, formaldehyde, or formate. Dihydrofolate reductase (DHFR) catalyzes a two-step reaction that converts folic acid to dihydrofolate (DHF) and then to THF.

Upon entering the folate cycle, THF is converted to 5,10-methylene THF (5,10-MTHF) by serine hydroxymethyltransferase (SHMT1) in the cytosol or by SHMT2 in the mitochondria [7]; this step concomitantly converts serine to glycine. Then, 5,10-MTHF is reduced further to 5-MTHF by methylenetetrahydrofolate reductase (MTHFR), which uses flavin adenine dinucleotide (FAD) as a cofactor. In a side pathway, 5,10-MTHF can act as a cofactor for thymidylate synthase (TS), which converts dUMPs to dTMPs for DNA synthesis and repair [13]. In a second major offshoot of this pathway, methylenetetrahydrofolate dehydrogenase (MTHFD) is a trifunctional enzyme that interconverts 5,10-MTHF (pyrimidine biosynthesis) and 10-formylTHF (purine biosynthesis) via a 5,10-methenylTHF intermediate [14].

Together, the folate cycle and the methionine cycle make up the one carbon metabolic pathway. The methionine cycle is essential for generating the universal methyl-donor of the cell, S-adenosylmethionine (SAM) [15,16]. The folate and methionine cycles intersect with 5-MTHF and methionine synthase (MS), which uses Hcy to generate THF and methionine. Methionine adenosyltransferase (MAT) is responsible for the hydrolysis of methionine to SAM. Methionine is an essential amino acid that is obtained from diet and is among the most variable in circulation [17]. Fluxes in the one carbon metabolism pathway regulate methionine and directly impact SAM concentrations, which in turn shape cellular signaling and epigenetics.

### 2.3. Vitamin B9 Regulation 

**Figure 3:**
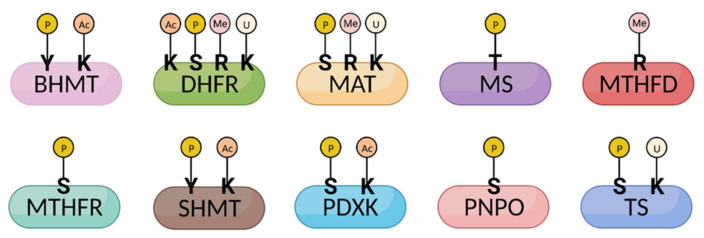
One-carbon enzymes are modified post-translationally. Enzymes involved in the one carbon metabolism pathway undergo a variety of post-translational modifications (PTMs), including phosphorylation, acetylation, methylation, and ubiquitylation. Several modifications (MTHFR phosphorylation and MTHFD1 methylation) affect protein activity and efficiency, thereby altering one carbon metabolic flux. Since identifying many other PTMs with proteomic screens, the biological effects of several other PTMs remain undefined. Note that the illustrated modifications only represent a selection of identified PTMs. Y, tyrosine; K, lysine; S, serine; R, arginine; T, threonine. P, phosphorylation; Ac, acetylation; Me, methylation; U, ubiquitylation. Figure made with BioRender.

The cell has evolved multiple levels of regulation to ensure the proper concentration of folate is maintained. Enzymes throughout the one carbon pathway are subjected to multiple forms of regulation through post-translational modifications, allosteric inhibition, and the abundance of key cofactors (Figure 3 and Table 1). The effects of post-translational modifications on one carbon metabolic enzymes have been recently investigated. 

Intrinsic feedback systems are essential for tuning the dynamics of one carbon metabolism. For example, MTHFR activity is sensitive to SAM levels. When present in excess, SAM allosterically inhibits MTHFR to reduce the regeneration of methionine; SAM levels of ~5 μM decrease MTHFR activity by half [35]. A phosphorylation of MTHFR by dual-specificity tyrosine phosphorylation-regulated kinase (DYRK1/2) primes a subsequent phosphorylation cascade by glycogen synthase kinase (GSK3) α/β that further sensitizes MTHFR to allosteric SAM inhibition [36]. Additional allosteric regulation occurs in the case of SHMT whose activity is inhibited by elevated THF levels above 40 μM [37].

Methylation senses SAM levels and dietary methionine. Since ATP, the cofactor for protein phosphorylation, is highly abundant in cells (~1–5 mM), kinase active sites are saturated by ATP because kinase Km, ATP is often in micromolar ranges [38,39,40]. Thus, the dynamics of substrate phosphorylation by kinases and phosphatases is independent of metabolism [15,41]. In contrast, alternative post-translational modifications that use less abundant metabolites as cofactors are rendered sensitive to the changes of cellular metabolic fluxes [42,43,44]. Methylation is paradigmatic of this phenomenon as the levels of methionine, the precursor of SAM, can rapidly change within minutes based on diet [17]. The impact of dietary methionine on cellular methylation was first appreciated in the regulation of epigenetics and the methylation of histones and DNA [45,46,47]. Similarly, demethylase activity relies on the abundance of a cofactor, α-ketoglutarate, which has been shown to be dynamically linked to canonical Wnt signaling [48]. More recently, methylation of lipids and proteins has also been shown to link the nutrient status of a cell with molecular signal transduction [47,49]. In fact, several B9- and other one carbon-metabolizing enzymes are methylated; in the case of MTHFD1, R173 methylation increases enzymatic activity and subsequently NADPH levels [27].

**Figure 4:**
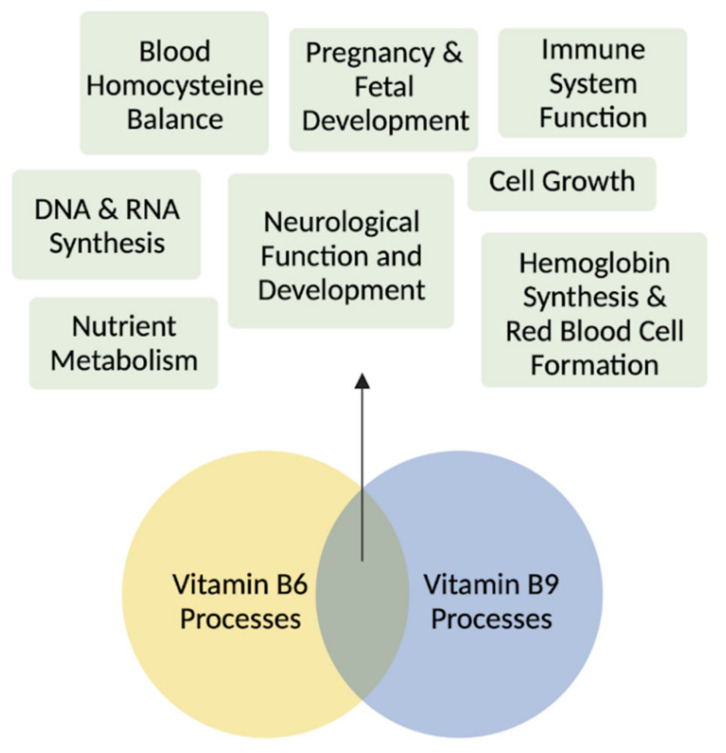
Vitamins B6 and B9 are essential in development and in adult tissues. Vitamin B6 and folate are involved in the biosynthesis of nucleotides for DNA and RNA synthesis, cell growth, neurological development during pregnancy and infancy, hemoglobin synthesis and red blood cell formation, blood homocysteine balance, immune system functioning, and nutrient metabolism. Figure made with BioRender.

A finely tuned crosstalk between vitamin B6 and folate plays central roles in several developmental and adult processes (Figure 4). Beyond the regulation by one carbon metabolites, folate levels are also regulated by other B vitamins such as B6 and B12. Active vitamin B6, PLP, is a necessary cofactor for enzymes linked to B9 metabolism (e.g., SHMT1/2 and cystathionine-β-synthase) and, therefore, altered B6 levels directly shape the fate of B9 vitamer conversions [50]. This regulation occurs for both SHMT isoforms, though it is interesting to note that cytosolic SHMT1 is more sensitive to decreased vitamin B6 than mitochondrial SHMT2 [51]. In the case of a vitamin B6 deficiency, folate cycling is disrupted due to the B6-dependent conversion of THF to 5,10-MTHF, thereby depriving neural cells of the amino acid precursors of several monoamine neurotransmitters, including dopamine and serotonin [52].

### 2.4. Folate Deficiency and Disorders

Folate deficiency (FD) has longstanding links to defective neurological and cardiovascular function [53], which can often be prevented by dietary supplementation. As 5-MTHF can be readily imported by somatic cells, it is often the recommended vitamer for B9 supplementation. A hallmark example of this condition is FD-induced spina bifida during embryogenesis, whose etiology is linked to hypomethylation and disrupted DNA mismatch repair [54]. Folic acid supplementation during the course of pregnancy can greatly prevent these defects; such recommendations from the WHO and FDA contributed to a 35–50% reduction in the incidence of FD-induced neural tube defects in the U.S. [55]. Beyond low folate diets, FD can arise through the loss-of-function mutations in key metabolizing enzymes such as MTHFR, where disrupted conversion of 5,10-MTHF to 5-MTHF alters DNA and purine synthesis, in addition to global methionine levels, resulting in impaired tissue development [28]. As a precursor for methionine synthesis, folate deficiencies are often congruent with altered methionine metabolism [56]. Alternative routes are available, however, to synthesize methionine from betaine and 5-MTHF via betaine-homocysteine methyltransferase (BHMT), although this route is limited to the liver and kidney [7]. In contrast, an excessive level of folate can in some cases be detrimental, such as through the masking of symptoms of vitamin B12 deficiencies, delaying diagnosis and therapeutic intervention [57,58].

### 2.5. Folate in Cancer

The folate cycle is intertwined with cellular programs that are integral to cancer metabolism and proliferation. Following the discovery of folic acid, Sidney Farber was inspired to test the hypothesis that folate could restore blood cells in leukemia, given its role in healing macrocytic anemia [59]. However, clinical trials abruptly stopped as folate was found to promote leukemia in patients. This unfortunate case led to the revolutionary concept of antifolate therapeutics, with methotrexate (MTX) being the first of its kind. Indeed, many folate enzymes are overexpressed in cancer. However, the roles of vitamin B9 during cancer cell initiation, transformation, and metastasis are highly dependent on the cancer type and surrounding tumor microenvironment (Table 2) [10].

Folate deficiencies have been linked to mechanisms of cancer progression [81]. Reducing folate cycling similarly decreases methionine cycling and SAM production, which may contribute to hypomethylation in cancer [82]. While cellular methylation dynamics on DNA and histone have been thoroughly studied, recent works highlight the significant impact of SAM homeostasis on the substrate methylation status of lipids, proteins, and RNA [47,49,83]. Additionally, folate deficiency can trigger DNA damage by decreasing the flux of dUMP to dTMP, which typically occurs during the TS-mediated side reaction that generates DHF from 5,10-MTHF. By increasing the dUMP:dTMP ratio, the rate of uracil misincorporation into DNA increases and could trigger single- or double-stranded breaks, and potentially oncogenic mutations [81,82]. Recent work showed that folate-supplemented diets protected mice prior to and during xenografting with pancreatic cancer cells [84].

#### 2.5.1. Lung Cancer

Methylenetetrahydrofolate reductase (MTHFR) has been linked to the progression of lung cancer, through the generation of fresh nucleotides for DNA synthesis and repair in the folate cycle [61]. Meta-analysis of MTHFR polymorphisms revealed that the most common variant in the MTHFR gene, C677TT, is correlated with an increased risk of lung squamous carcinoma in East Asian populations [60,85]. Surprisingly, the 677TT mutation reduces MTHFR activity by 70% by disrupting FAD cofactor binding [86]. A reduction in MTHFR efficiency lowers folate in circulation, decreasing methionine and SAM levels, and causing an accumulation of Hcy. Notably, high Hcy is a risk factor in cancer and cardiovascular disease by increasing red blood cell coagulation and pro-inflammatory pathways [87,88,89]. Individuals with this variant have a folate blood concentration 16% lower than those with the 677CC genotype, indicating that high levels of B9 have also been associated with reduced risk of lung cancer [90]. Interestingly, a hospital-based case-control study of a non-Hispanic white population found that the 677TT genotype was associated with a decreased risk of lung cancer in women but not in men [61]. Dietary vitamin B6, vitamin B12, and methionine in women with C677T genotypes were associated with a decreased risk of cancer. Conversely, the MTHFR 1298CC genotype was associated with an increased risk of lung cancer in women [61].

#### 2.5.2. Colon Cancer

Low folate intake has been associated with an increased risk of colon cancer [91] and long-term folate supplementation was shown to lower the risk of colon cancer by 75% [92]. Folate has been implicated in colorectal carcinogenesis through mechanisms of DNA synthesis and methylation [93]. As the folate and methionine cycles are at the center-stage of one carbon metabolism, low levels of folate lead to decreased methionine in circulation and a reduction in DNA methylation essential for DNA expression, stability, and repair [94,95]. Folate cycle intermediates acting as cofactors for purine and pyrimidine synthesis are also essential for DNA synthesis [96,97]. Due to this, low levels of folate have been associated with aberrant DNA regulation and stability via strand breaks, mutations, and hypomethylation [96,98,99,100]. Human colonocytes cultured in folate-deficient media were unable to repair DNA strand breaks and showed a five-fold increase in uracil misincorporation. Proteomics revealed altered activity and expression of proteins involved in proliferation, DNA repair, apoptosis, and malignancy in these cells [101]. Folate deficiency was also shown to induce mitotic aberrations [102]. A meta-analysis investigating the relationship between folate supplements and colon cancer risk found an inverse correlation between the two [62]. Similarly, folate deficiency has been shown to aggravate carcinogenesis in colon cancer rat models, while increasing dietary intake reduced neoplasms [63,64]. Nevertheless, epidemiological studies have failed to find an association between dietary and circulating folate and colorectal cancer risk [65,66].

#### 2.5.3. Pancreatic Cancer

Similar to colorectal cancer, molecular underpinnings between pancreatic carcinogenesis and folate appear to involve DNA hypomethylation and impaired DNA synthesis, but specific insights are yet to be uncovered. Conclusions regarding dietary folate intake yielded inconsistent results [71]. A meta-analysis investigating the link between folate intake and MTHFR polymorphisms revealed that the 677TT variant was associated with increased risk of gastrointestinal and pancreatic cancer. This variant lowers circulating folate by preventing 5-MTHF synthesis from 5,10-MTHF, which reduces DNA methylation. In contrast, meta-analyses based on folate intake instead of genetic mutations revealed a decreased risk of pancreatic cancer [72,73]. Together, this suggests that MTHFR status may dictate the relationship between folate and pancreatic cancer prognosis.

#### 2.5.4. Ovarian Cancer

Ovarian cancer is the most lethal cancer of the female reproductive system [103]. Interestingly, 80% of ovarian cancers have an overexpression of folate receptors (FR), which is largely absent in healthy tissue. Thus, FRα serves as a serum biomarker [104] in ovarian cancers. Folate receptor α (FRα) binds the active form of folate and transports it inside cells via receptor-mediated endocytosis. This receptor overexpression leads to a higher intake of folate into the cell, increasing rates of DNA synthesis that facilitate cancer cell growth. This receptor overexpression on cell surfaces has allowed FRα to emerge as an attractive target for monoclonal antibody therapies such as farletuzumab. Though epidemiologic studies report no association or an inverse association between folate intake and ovarian cancer risk [67,70], FRα has been shown to increase chemotherapy resistance by stabilizing murine double minute 2 (MDM2), an oncogene that can be used as a prognostic factor in ovarian cancer [68,69].

#### 2.5.5. Esophageal, Liver, and Gastric Cancer

A systematic meta-analysis of esophageal cancer found a decreased cancer risk within a certain folate intake range [105]. Accordingly, vegetarian diets have been shown to be protective against esophageal cancer [106]. Just as FRα is overexpressed in ovarian cancer, tumor-associated macrophages weaponize folate receptor β (FRβ) to promote liver cancer metastasis [107]. While folate levels are not increased in hepatocellular carcinoma, FRβ can serve as a potential therapeutic target [108] through FRβ-targeting lipid nanoparticles that deliver anti-neoplastic drugs [109]. Folate deficiency is a risk factor for gastric cancer. Interestingly, gastric cancer is frequently related to vitamin B deficiencies such as B12 in pernicious anemia. Whether folate drives gastric cancer or is merely a byproduct of oncogenic metabolism remains an open area of research [110]. 

#### 2.5.6. Prostate Cancer

Epidemiologic studies have reported inconclusive correlations between folate and prostate cancer prognosis. Two meta-analyses found no association between folic acid and prostate cancer risk [74,75], while one meta-analysis found a 24% increase in risk [76]. Additionally, a case-control study found that low levels of folate and high levels of Hcy were associated with various cancers including prostate [78]. Another study found a 4% increase risk with every 5 nmol/L increase in serum folate, although dietary folate intake had little to no effect on cancer risk [77]. During prostate cancer, polyamine levels required for normal prostate growth are increased [111,112]. High levels of polyamines have been shown to sensitize cells to folate. Conversely, inhibition of adenosylmethionine decarboxylase 1 (AMD1) blocks polyamine synthesis, increasing SAM and decreasing folate levels in the cell [113]. This raises the possibility that increased folate sensitivity increases the rate of DNA synthesis, driving prostate cancer progression.

#### 2.5.7. Breast Cancer

A systematic review and meta-analysis found a U-shaped relationship between folate concentration and breast cancer risk, where women with dietary folate intake between 153 and 400 μg showed reduced breast cancer risk, unlike those outside of this range. No correlation was found with circulating folate levels [79]. It is reported that the usual folic acid dosage for breast cancer patients is less than 400 μg per day [114]. Notably, the chemoprotective effect of folate in this study was more pronounced in women with high alcohol consumption, as alcohol is an established breast cancer risk factor [115]. Alcohol is a known antagonist of folate and interferes with folate metabolism by disrupting uptake, storage, and release from hepatocytes [116]. Folate supplements are recommended to reduce cancer risks associated with alcohol use. Nevertheless, other studies report no influence of blood or dietary folate levels on breast cancer risk [72,79,80].

## 3. Vitamin B6: Pyridoxine

### 3.1. Dietary and Active Forms

Vitamin B6 has been considered to be the forgotten B vitamin as the clinical manifestations are less severe than B9 deficiencies. However, vitamin B6 is an essential cofactor with numerous regulatory functions in glycogen catabolism, gluconeogenesis, lipid metabolism, amino acid synthesis, heme biosynthesis, neurotransmitter biosynthesis, anaplerosis, and redox homeostasis [117,118]. It can be directly obtained from diet via fish, beef liver, fortified cereals, dark leafy greens, chickpeas, and potatoes. PLP and PMP are the primary derivatives found in animal-derived foods, while plant-derived foods are primarily PN, PNP, and a modified PN, pyridoxine-5′-β-D-glucoside (PNG) [52,119]. The physiological outcomes of these molecular pathways serve to regulate many tissues. Initially, vitamin B6 was discovered as an anti-dermatitis factor by Paul Gyürgy in rats on a riboflavin and thiamin diet that developed acrodynia [120]. Recommended vitamin B6 doses have been clearly defined as 1.3 mg/day, or 1.9 mg/day during pregnancy to support neural development [121]. Supplements can reach over 5000% of the required daily value. Regardless of this overabsorption, the majority of the supplemented vitamin B6 is excreted in urine as 4-pyridoxic acid (PA) [122]. Notably, high-performance athletes may benefit from vitamin B6 supplements as physical exercise increases the excretion of PA [123], with a study reporting lower B6 levels in endurance athletes after exercise [124]. Plasmatic PLP can increase 10-fold upon supplementation and respond within 1–2 weeks following depletion or repletion. PLP also decreases within hours of carbohydrate ingestion [125,126]. Interestingly, studies have shown that although plasmatic PLP in an individual fluctuates with B6 intake, cellular and tissue PLP levels remain relatively steady [127]. Vitamin B6 levels in patients can be approximated by levels of PLP and Hcy in plasma, levels of PLP in erythrocytes and blood, the urinary excretion of 4-PA, and tryptophan catabolites [121].

### 3.2. Metabolic Pathways and Key Enzymes in Pyrixodine Metabolism

**Figure 5:**
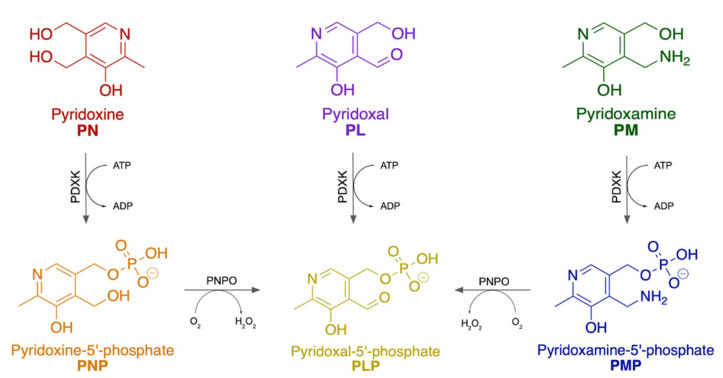
Vitamin B6 is an umbrella term for various vitamers. Vitamin B6 is ingested as the vitamer pyridoxine (PN, red) and is phosphorylated by PDXK to become pyridoxine-5′-phosphate (PNP, orange). PNP is finally converted to the most metabolically active form of vitamin B6, pyridoxal-5′-phosphate (PLP, yellow), via PNPO. PLP can also originate from pyridoxal (PL, purple) via a one-step phosphorylation reaction by PDXK, or from pyridoxamine (PM, green) via a two-step enzymatic reaction by PDXK and PNPO, where it is first phosphorylated to become pyridoxamine-5′-phosphate (PMP, blue) before becoming PLP.

Structurally, B6 is a substituted pyridine with a hydroxyl and methyl group at the 5 and 6 positions, respectively (Figure 5). The 4 position is interconverted between a hydroxymethyl, formyl, or amino group at different stages in the metabolic pathway; another hydroxymethyl group at the 2 position can be phosphorylated. These variations account for the B6 vitamers: pyridoxine (PN), pyridoxal (PL), pyridoxamine (PM), and their phosphorylated equivalents, pyridoxine-5′-phosphate (PNP), pyridoxal-5′-phosphate (PLP) and pyridoxamine 5′-phosphate (PMP). Vitamin B6 is ingested as PN and is converted to PNP via pyridoxal kinase (PDXK), an ATP-dependent enzyme. Then, PNP is transformed to PLP by pyridoxine 5′-phosphate oxidase (PNPO). Both PL and PMP can be directly phosphorylated to PLP or PMP, respectively, via PDXK. Finally, PMP is converted to PLP by PNPO. The active B6 vitamer is PLP, which is the dominant form in circulation and accounts for 60–70% of B6 in humans, while PL is secondary at 30% [128]. 

In physiology, PLP is a cofactor for over 160 enzyme reactions [129]. As the dominant vitamer, PLP is an index for general B6 measurements; however, PL and 4-PA are also measured, usually by employing fluorometric high-performance liquid chromatography (HPLC) or liquid chromatography-tandem mass spectroscopy (LC-MS/MS) [130]. Some studies have suggested measuring total vitamin B6 or at least PLP+PL in order to (1) minimize person-to-person variability and (2) account for different levels of albumin (binds PLP in the bloodstream) and alkaline phosphatase (AP, converts PLP to PL) [131,132].

### 3.3. Vitamin B6 Regulation

Vitamin B6 is absorbed in the jejunum, metabolized to its active form in the liver, and excreted in urine from the kidney. Pyridoxal kinase (PDXK) is expressed in many tissues, with its highest expression being in the cerebral cortex and basal levels in the adrenal gland, lung, breast, gastrointestinal tract, urinary tract, testis, and adipose tissue [133]. As a main regulator of PLP levels and B6 activity, PDXK regulation occurs at the transcriptional and post-translational levels to shape PLP flux. Several phosphorylations decorate PDXK (multiple of which are mutated in cancer patients), although the effects of these post-translational modifications (PTMs) have yet to be defined [20,31,134] (Figure 3). To restrict PDXK enzymatic output, PDXK can be tagged for proteasomal degradation at eight identified ubiquitination sites [135]. It can also be pharmacologically inhibited with drugs such as 4′-O-methylpyridoxine, which competitively inhibits the PDXK active site [136]. Beyond vitamer interconversion, B6 activity can also be suppressed via PLP sequestration by a PLP-binding protein [137]. Natural PN antagonists such as 1-amino-D-proline exist in foods such as flaxseed, which reduce PLP levels and the output of several PLP-dependent enzymes [138].

Cellular uptake requires PLP dephosphorylation by a membrane-bound AP, which is expressed across several organs (bone, kidney, and liver) [81]. PLP and PMP are the primary derivatives found in animal-derived foods, while plant-derived foods are primarily PN, PNP, and a modified PN, pyridoxine-5′-β-D-glucoside (PNG) [52,119]. Interestingly, a study reported a 13% loss of vitamin B6 in food after cooking, though the remaining vitamin B6 was still sufficient to meet daily requirements [139]. Vitamin B6 is also available as supplements as either a complex with other B vitamins or as PN (specifically as pyridoxine hydrochloride).

### 3.4. Pyridoxine Deficiencies and Disorders

Vitamin B6 deficiency is caused by reduced intake, poor absorption, or increased utilization. Low intake of vitamin B6 has been associated with malnutrition, whereas low absorption can be caused by alcoholism where PLP fails to be released from the liver [140]. In areas with adequate access to food, diet-based B6 deficiencies are less common and are instead exacerbated by environmental factors [141], smoking, and adverse drug interactions [142,143,144]. Increased demand for vitamin B6 is common in pregnancy, where it promotes central and peripheral nervous system development [145]. Beyond its role as a cofactor for over 160 enzymes, B6 deficiency alters neurological functioning via depressed N-methyl-D-aspartate (NMDA) receptor function [145]. Medications such as isoniazid can also interfere with vitamin B6 metabolism. Isoniazid is used to treat tuberculosis but can lead to peripheral neuropathy by interfering with the metabolism of PN, an essential component of the nervous system. Isoniazid reacts with PL to form hydrozone derivatives that act as a PDXK inhibitor, therefore reducing PLP levels required for central nervous system function [146,147].

Vitamin B6 levels directly impact a wide array of physiological processes and deficiencies can present as pruritic rash, glossitis (tongue swelling), cheilitis (cracks on lips and skin), and neurological disorders [148]. Despite divergent manifestations, many of these disorders converge with mechanisms of poor antioxidant defense, lower purine synthesis, and disrupted glutathione synthesis. Severe deficiency can result in dermatitis, where B6-dependent enzymes involved in creating collagen amino acid precursors lack their necessary cofactor [149], and anemia, as low levels of PLP hinder heme synthesis. Other consequences of low vitamin B6 are nausea, confusion, depression, and even dream loss [150]. 

Similar to folate, vitamin B6 is critical for neurological function and red blood cell formation. The neuroprotective role of B6 stems from its capacity as a cofactor for glutathione, thereby lowering the aging brain’s susceptibility to oxidative stress-based damage [151]. Thus, B6 deficiencies directly skew oxidative stress balance, thereby contributing to neurodegeneration as cells accrue damage from reactive oxidative species [152]. In the bone marrow, PLP is used as a coenzyme in heme synthesis and subsequently hemoglobin formation [153]. Interestingly, Parkinson’s patients treated with L-DOPA often report vitamin B6 deficiency, which can induce anemia [154]. The classic vitamin B6 antioxidant role has also been shown to support diabetes prognosis. Diabetic rats treated with vitamin B6 showed marked reductions in oxidative stress markers in tissues [155,156]. Notably, the role of vitamin B6 as an antioxidant also allows for the quenching of reactive oxygen species resulting from UV light [157].

In addition to diet, low PLP can also result from genetic mutations in PDXK, PDXP, or PNPO enzymes [119]. Loss-of-function mutations in PDXK are found in Charcot-Marie-Tooth disease, which could be remedied by PLP supplementation, and PNPO enzyme mutations have been reported in early onset epileptic encephalopathy [158,159,160]. 

Conversely, high levels of vitamin B6 also contribute to pathologies across different tissues. Most notably, excess PN, both from PNPO mutations as well as supplement abuse, has been linked to sensory neuropathy characterized by numbness, nerve damage, and muscle pain [161]. Interestingly, a cohort study of post-menopausal women revealed that those treated with high doses of vitamin B6, a common treatment of menopausal symptoms, had an increased risk of hip fractures [162]. Similarly, vitamin B6 has recently emerged as a novel biomarker for ankle fractures and osteoporosis, where high levels of vitamin B6 are thought to overstimulate bone remodeling and erode bone tissue [163]. Additionally, pellagra, a B3 deficiency characterized by scaly skin, has been reported to be aggravated by high levels of vitamin B6 [164]. Several other mechanisms of B6 involvement in disease include immune suppression by downregulating pro-inflammatory cytokines such as IL-1β and IL-18, thereby shutting down NF-κβ and JNK activation, and suppressing the NLRP3 inflammasome [165].

### 3.5. Pyridoxine and Cancer

The physiological roles of vitamin B6 in coordinating cellular metabolism and amino acid synthesis are misregulated in numerous cancers. Vitamin B6 was first linked to cancer in the late 1960s when increased urinary excretion of tryptophan in Hodgkin’s lymphoma patients was related to plasma PLP deficiency, as PN deficiencies are known to impact the metabolism of amino acids including L-tryptophan [51]. Since this discovery, vitamin B6 has been implicated across a spectrum of cancers in tissues derived from the colon, lung, breast, and blood. Intensive efforts have focused on distinguishing which cancers are exacerbated or shunted by vitamin B6 supplementation. The multifaceted roles of vitamin B6 in cancer and recent mechanistic insights are detailed in Table 3.

#### 3.5.1. Colorectal Cancer

High levels of vitamin B6 have been correlated to a reduced risk of colon cancer [167] in population-based studies as marked by plasma PLP concentrations. A population-based study revealed that a diet high in vitamin B6 reduced the risk of alcohol-associated colon cancer in women that consumed moderate and high amounts of alcohol, as alcohol leads to decreased vitamin B6 by interfering with methionine synthase and vitamin B6 synthesis and absorption [168]. 

In a xenograft model of colorectal carcinoma, vitamin B6 metabolism was among the most upregulated in an exercise-induced model [170]. Additionally, mechanistic insight into the role of vitamin B6 in colorectal cancer cell lines found that PL significantly stimulated p21 mRNA expression, which is well known as a negative regulator of the cell cycle [185]. Similarly, PL raised phospho-p53 levels, which promote p21 expression, though no changes in p53 protein levels were observed. These results were recapitulated in mice, where mice fed diets low in vitamin B6 displayed low expression of p21 mRNA. As p21 and p53 are known to suppress cell proliferation, vitamin B6 levels may play a role in the p21 and p53 pathways leading to tumorigenesis [169].

#### 3.5.2. Pancreatic Cancer

A meta-analysis examining the association between vitamin B6, vitamin B12, methionine levels, and risk of pancreatic cancer revealed that increasing concentrations of vitamin B6 inversely correlated with risk of pancreatic cancer. Similarly, increasing blood PLP levels also showed an inverse association with pancreatic cancer risk, with risk decreasing by 9% for every 10 nmol/L increment [178]. Consistent with this, a Singaporean–Chinese health study revealed that at a 16-year follow-up, 271 pancreatic cancer cases were identified out of over 63,000 men and women enrolled, with a higher intake of vitamin B6 statistically correlating with a decrease in pancreatic cancer risk [186].

#### 3.5.3. Lung Cancer

Lung cancer is the most common cause of cancer death in all sexes [187]. Similar to colorectal cancer, high levels of vitamin B6 have also been reported to reduce lung cancer risk. In non-small cell lung cancer (NSCLC) patients under cisplatin regimens, vitamin B6 sensitizes cancer cells to cisplatin-mediated DNA damage and cell apoptosis [171]. Low PDXK levels also correlated with poor disease prognosis. Consistent with this, a case-control study revealed that among diverse participants, high vitamin B6 serum levels correlated with a decreased risk of lung cancer [172]. It should be noted, however, that contradictory studies showed correlations between high vitamin B6 levels and high incidence of lung cancer, and vice versa [173,174]. Nevertheless, there have also been reports finding no association between sera and dietary B6 levels with increased cancer risk in lung tumor sites [175].

#### 3.5.4. Breast Cancer

Breast cancer is the most common cancer in women (cancer.gov). Unlike the established role of vitamin B6 in cancers such as that of the colon and the lung, there is a less convincing association between vitamin B6 and breast cancer prognosis. Population-based studies have reported different results. A 2001 study reported that breast cancer patients presented with higher serum vitamin B6 levels than the control group [176], while a 2004 study reported no correlation between breast cancer risk and vitamin B6 intake or serum PLP levels [177]. A study on the link between folate and breast cancer risk revealed that vitamin B6 increased the protective effect of folate on breast cancer, lowering risk [176]. This effect suggests that different players of the one carbon metabolism may also collaborate in preventing or reducing cancer incidence. It would be interesting to evaluate how folate-based dietary interventions combine with methionine-related chemotherapeutics, such as methyltransferase inhibitors like MS023, which reduces triple negative breast cancer development [188].

#### 3.5.5. Prostate Cancer

Prostate cancer is the second most common cancer among men (cancer.gov). A meta-analysis of observational and interventional studies assessing the association between vitamin B6 intake, PLP levels, and cancer risk revealed that there was no statistically significant association [175]. Nevertheless, a previous study observed that an increasing consumption of vitamin B6 reduced risk [180], and suggested it could be due to the role of vitamin B6 in organ sensitivity to hormone uptake and action, which is increased with low levels of the vitamin [189].

#### 3.5.6. Skin Cancer

Though vitamin B6 has been shown to have protective properties on the skin, such as its anti-oxidant and anti-inflammatory roles, there is little to no evidence linking it to cancers of the skin, warranting more clinical and scientific attention. A 1985 in vitro study revealed that B16 melanoma cells grown with 5.0 mM PN or 0.5 mM PL had an 80% reduction in cell proliferation [181]. Furthermore, B16 melanoma xenografted mice pretreated with PL showed a 62% reduction in tumor growth compared to those without the pretreatment. Similarly, a 2018 study revealed that PL at 500 μM suppressed B16F10 cell proliferation, while PN had a weak inhibitory effect [182], with both studies supporting that PL has a stronger inhibitory effect on melanoma cell growth at lower concentrations compared to other B6 vitamers. Though there appears to be potential, whether vitamin B6 may serve as an antineoplastic drug for skin cancers requires further investigation.

#### 3.5.7. Kidney Cancer

Vitamin B6 is part of the vital renal vitamins, and is commonly supplemented to Chronic Kidney Disease (CKD) patients at 5 mg/day on non-dialysis and 10 mg/day on dialysis regimens [190]. The role of vitamin B6 in the context of renal cancer remains to be closely investigated. A World Cancer Research Fund-funded study revealed that patients with higher plasma concentrations of vitamin B6 had reduced risks of renal cell carcinoma and overall better prognosis [183]. In support of this, a case-cohort study revealed that higher vitamin B6 concentrations were associated with lower risk of death in renal cell carcinoma patients [184]. Similarly, PLP depletion in diabetic patients has been linked to an increased risk of developing several malignancies, including that of the kidney [191], though a meta-analysis study revealed no evidence of association between high vitamin B6 intake and kidney tumors [175].

#### 3.5.8. Acute Myeloid Leukemia

Vitamin B6 was originally thought to play a role in blood cancers, such as leukemia. This was attributed to the role of vitamin B6 in red blood cell production [192]. Following this discovery, low levels of vitamin B6 have been continuously reported in blood cancers, where low levels are correlated with worse outcomes. In the context of acute myeloid leukemia (AML), high vitamin B6 levels are associated with increased risk of cancer. Vitamin B6 is low in circulation of AML patients, which may indicate an addiction of AML cells to vitamin B6 [136,166]. In normal tissues, PDXK phosphorylates inactive into active vitamin B6 when cellular levels are low. In AML cancerous states, PDXK constitutively phosphorylates PL and PN into PLP, thereby promoting cancer cell proliferation, while a dominant negative PDXK mutant was unable to restore PLP levels or the proliferative effects of AML cells [136]. These surprising findings were uncovered using a CRISPR-Cas9 screen to determine which metabolic enzymes required PLP for AML proliferation, revealing GOT2 and ODC [136], which may also support dysregulated cell growth by increasing one carbon metabolic flux towards biomacromolecule precursor synthesis. This deeply mechanistic experimental approach provided new insights into a central mystery in the field of vitamers and oncology.

#### 3.5.9. Brain Cancer

It is interesting to note the absence of vitamin B6 involvement in brain-related cancers given the high levels of PDXK expression across neuronal tissues, with the exception of some neuroblastoma studies [193]. One could speculate that high PDXK may be chemoprotective by promoting PLP production.

## 4. Key Advances and Implementation of Innovative Tools and Instrumentation

Vitamin B6 and B9 levels have been historically measured Ex vivo from blood or sera samples using microbiological or enzymatic assays [194,195]. However, approximating one carbon metabolite levels within cells was limited until recent advances of metabolomics-based mass spectrometry [196]. An additional challenge surrounds the measurement of metabolic fluxes, particularly in real time, where the metabolism within a cell can dramatically change within seconds [197]. Modeling metabolic flux in vitro with cell lines also suffers from current culture methods, where excess nutrient levels can skew the metabolism of a cell [198]. Exciting advances using in vivo imaging, physiologic cell culture media, metabolite labeling, and organelle proteomics have resulted in new discoveries surrounding one carbon metabolism in physiology and pathological states. Indeed, metabolomics enables the precise quantification of intracellular metabolites with resolution to distinguish between different vitamers [199,200,201,202,203]. When combined with metabolite labeling in vivo, these advances have enabled metabolic flux measurements and paradigm-shifting insight for the fields studying folate and PN metabolism [204].

Metabolomics has also offered novel biomarkers and new areas for therapeutic intervention in researching the etiologies of B6- and B9-related disorders. In the case of PN-dependent epilepsy, untargeted metabolomics uncovered several candidate biomarkers in blood (α-aminoadipic semialdehyde, piperideine-6-carboxylate, and pipecolic acid) that had been previously disregarded given their instability in urine-based sampling [205]. Given the higher sensitivities of modern analytical tools, clinical sampling is more practical as in the case of cerebrospinal fluid detection of several B6 vitamers [206,207]. In vivo metabolite measurements overcome major obstacles in the field by correlating metabolic activity with gene expression and patient histology. A particularly exciting innovative technology overcomes these limitations and even enabled quantitative metabolic flux monitoring of human tumors. The De Bernardis lab applied multiparametric, preoperative imaging with intraoperative infusions of isotope-labeled nutrients (e.g., ^13^C-glucose) to resolve the dynamics of metabolism in lung and brain tumors and even across organ systems [208]. Metastasis rates in a profile of 17 patient-derived xenograft melanoma mouse models were directly linked to variable usages of methionine metabolism, which was explained by H3K9 and H3K27 methylation patterns [27]. Modulating the levels of methionine in the diets of cancer patients has become of increasing clinical interest, where methionine usage directly explains features such as metastatic potential [27,209]. Since B6 and B9 both control one carbon metabolic flux, combining B6 and/or B9 restrictions with methionine restriction therapies could offer synergistic benefits for patient health. Use of new techniques that can decipher the differences between the tumor metabolic environment from those in adjacent and benign tissues could provide insight into new vulnerabilities for targeting cancer tissues specifically. 

Additional transformative techniques include those to visualize metabolite levels in vivo or in vitro. Folate uptake by cells has historically been imaged using radiolabeled or fluorophore-conjugated folate analogs [210]. Such techniques have progressed to the point of observing fluorescence in operating rooms, as is the case with Cytalux. Cytalux is a fluorescent folate analog that was approved by the FDA in 2021 to illuminate cancer lesions for diagnosis, as well as during surgery for immediate identification of malignant versus benign tissue [211]. Cytalux binds to folate receptors, which are overexpressed in ovarian and lung cancers, and can be excited via near-infrared light [210]. 

Imaging B6 has proved elusive until a first-of-its-kind probe was developed by Jun et al. in 2019. Utilizing the reactivity of acyl-hydrazides with aldehydes, they designed a rhodamine-based probe that selectively fluoresces upon binding PLP [212]. The probe demonstrated excellent selectivity among other biological aldehydes—including other B6 vitamers. The probe was employed in vitro to monitor the conversion of PL to PLP upon addition of PDXK, with fluorescence building over an 18-h time course. The probe was further tested in control and PDXK-knockdown cells. Interestingly, PLP levels in PDXK-deficient cells remained similar to that of control cells, hypothesized to be a result of PNPO conversion of PMP and PNP to PLP. However, the addition of exogenous PL specifically enhanced fluorescence. Another B6 probe was recently developed by Brun et al. in the form of a hydrazine-derived contrast agent (2-hydrazinonicotinic acid, or 2-HYNIC) that reacts with an aldehyde to form a hydrazone in 2021 [213]. 2-HYNIC adopts a unique planar conformation when bound to PLP, allowing for enhanced contrast and hence imaging using chemical exchange saturation transfer MRI (CEST-MRI). With this technology, B6 metabolism was tracked in vitro in lung cancer cell lines, as well as in vivo with animals bearing tumor xenografts. Differences in CEST-MRI contrast between the cell lines reflected metabolic differences of tumor cells [171].

Nucleic acid-based imaging modalities likewise offer a unique approach that could promote the rapid development of selective metabolite sensors for vitamins B6, B9, and their constituent vitamers. Aptamers are short nucleic acid sequences that bind a ligand, and thus can be harnessed for use as sensors. Upon binding to a target of interest, aptamer sensors fold into a structure that resembles the fluorophore of fluorescent proteins [214,215]. Aptamer sensors have already been used and optimized to detect metabolites in one carbon metabolism, including SAM [216,217]. While fluorescent aptamers are now well-characterized, more difficulty lies in designing a high-affinity ligand-binding sequence. Conveniently, bacteria contain endogenous small-molecule-binding RNA regulatory elements among their mRNA known as riboswitches. A riboswitch has been discovered for THF [214,218,219]. In principle, the appropriate riboswitch could be coupled to a fluorescent aptamer, granting dynamic imaging of folate metabolism. It is predicted that thousands of other riboswitches exist for metabolites, which may enable complex imaging of the metabolic interplay between B6, B9 and other metabolites [220]. Other imaging approaches have exploited inducible CRISPR-dCas9 transcription activation of a fluorescent protein reporter combined with a split riboswitch system that associates upon SAM binding [221], or riboswitches for ZTP, a molecule that indicates a lack of folate biosynthesis [222].

As long-awaited goals of sensing small molecules in living contexts have been realized and continue to advance, it will be easier to illuminate these metabolic mechanisms to advance biomedical sciences. The complementation of these sensors can further expand the chemical toolbox to improve understanding of one carbon metabolism and pathogenicity.

## 5. Exploiting One Carbon Metabolism for New Therapeutics

Metabolic enzymes across the folate and methionine cycles have been shown to be overexpressed in cancer. Recent advances have already led to exciting new therapies that target key enzymes in the one carbon pathway. Advances in technology have allowed deeper mechanistic insight and offer a vulnerability for designing novel therapies.

The DHFR enzyme is the target of MTX, the first antimetabolite therapeutic that was initially developed for the treatment of acute lymphoblastic leukemia (ALL) [58]. The success of MTX-based interventions gave rise to a series of antifolate cancer therapies such as 5-fluorouracil (5-FU) [223], which targets TS in the folate cycle. MTX has been prominently used to treat psoriasis, rheumatoid arthritis, and several cancers [72,224,225]. MTX functions through several mechanisms related to inflammation and proliferation; within the context of cancer, its inhibitory effects on DHFR block conversion of DHF to THF, thereby slowing metabolic flux towards nucleotide synthesis and halting proliferation. Challenges surrounding toxicity limit the extended use of MTX treatments, depleting THF pools from healthy cells [226]. A CRISPR-Cas9 screen of MTX-treated leukemia cells revealed a possible mechanism to attenuate MTX-induced cellular toxicity of healthy cells. Formimidoyltransferase cyclodeaminase (FTCD) is an enzyme that uses THF as a cofactor to perform histidine catabolism. In the presence of histidine supplementation, available pools of THF were redirected to the histidine catabolic pathway, increasing the sensitivity of cells to MTX and reducing its toxicity [227,228]. This landmark discovery highlights the fundamental importance of environment folate sources to antifolate therapies and provides mechanistic insight into improving therapeutic conditions. Indeed, several exciting reports have followed this and similarly demonstrate how cancer cells overexpressing folate pathway enzymes can be targeted with higher specificity. Either MS or MTR convert 5-MTHF, the predominant form of folate in circulation, to THF and are overexpressed in many cancer cell types. Exposing cancer cells to dietary 5-MTHF was sufficient to increase resistance to MTX-based treatments [229,230].

Small-molecule SHMT inhibitors have recently been shown to block the growth of many human cancer cells [231]. Cancers with defective amino acid transport systems such as B-cell lymphoma are particularly vulnerable to SHMT inhibitors because glycine is the byproduct of SHMT reactions. MTHFD2 is overexpressed in cancer and contributes to genomic instability during the early stages of cancer initiation that are associated with an activated DNA-damage response (DDR) [50]. MTHFD2 inhibitors prevented thymidine production leading to misincorporation of uracil into DNA and replication stress. 

Tumor cell metabolism is rewired to enable aberrant proliferation and expansion [232]. However, cancer cells do still rely on metabolites provided by the patient’s diet. For this reason, dietary restriction of specific amino acids has been deeply investigated as a potential therapy across different cancer types. Methionine is a prime example of this new avenue of research [233]. In KRAS-driven lung and pancreatic cancers, reduction of asparagine showed promising results [234]. Within the one carbon pathway, limiting dietary methionine reduced tumorigenesis in chemotherapy-resistant KRAS-driven colorectal cancer and radiation-resistant KRAS-driven soft tissue sarcoma [235,236]. Similarly, lowering the levels of non-essential amino acids serine and glycine was sufficient to reduce tumor progression in APC-inactivated models of intestinal cancers [237]. It is interesting to note that cancer cells may use 50% of glucose-derived carbon for serine biosynthesis and catabolism [215]. The amplification of breast and melanoma cancers has an upregulation of 3-phosphoglycerate dehydrogenase (PHGDH), a rate-limiting serine biosynthesis enzyme that was found to be required for growth in vitro and in vivo models [238,239].

**Figure 6:**
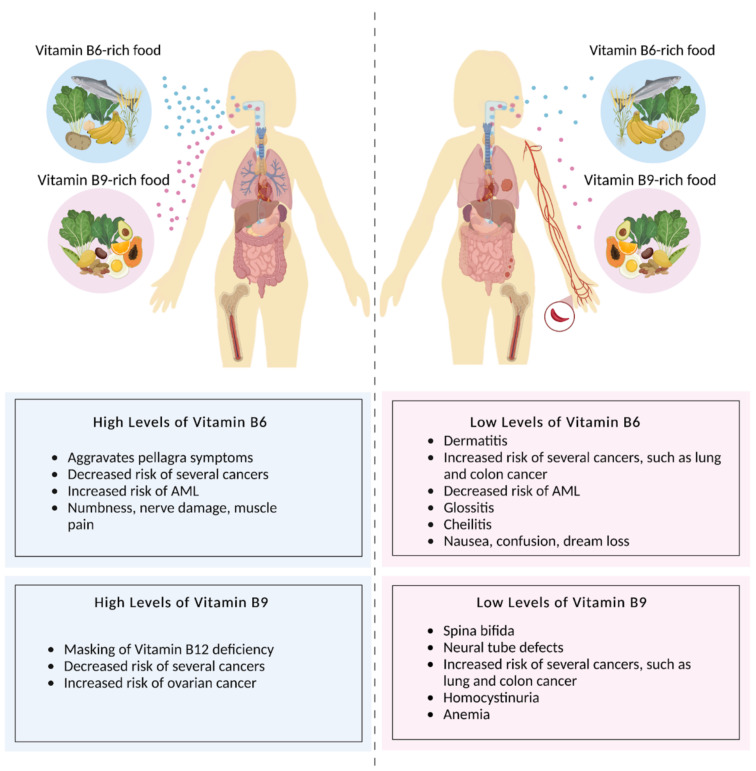
Dietary vitamin B6 and folate are essential for human health. Diets high in vitamin B6- and B9-rich foods (left) correlate with decreased risks of several cancers, but may mask the symptoms of a vitamin B12 deficiency, increase risk of ovarian and AML cancers, aggravate pellagra symptoms, and lead to numbness, nerve damage, and muscle pain. Diets low in vitamin B6- and B9-rich foods (right) can lead to dermatitis, glossitis, cheilitis, nausea, confusion, dream loss, as well as an increased risk of several cancers and a decreased risk of AML. Figure made with BioRender.

The combination of metabolomics and metabolite tracing analyses led to the discovery that tumor-initiating cells have high activity of the methionine cycle via MAT2A overexpression. Importantly, the high consumption of methionine outcompetes regeneration and renders these cancer cells auxotrophic to methionine and vulnerable to pharmacological inhibition [240] (Figure 6). Similarly, genetic mutations in one carbon metabolic enzyme methyl-5′-thioadenosine phosphorylase (MTAP) offer a point to help identify tumor cell populations that could be vulnerable to reducing dietary methionine [241,242,243,244]. The mechanism for this underlies the methionine salvage pathway and the generation of metabolite MTA, which acts as an inhibitor of methylation. Reports from multiple labs have now shown that MTA accumulation with either methionine deprivation or methylation inhibitors led to dramatic decreases in proliferation of many cancers. This exemplifies how genetics can help to inform clinicians in the design of dietary regimens during chemotherapeutic interventions [17]. As metabolic disease states cannot be determined using classic genomic analysis, these lines of research elucidating the contributions of metabolic regulation and enzyme mutations in disease will provide key insight for clinicians and basic researchers.

## 6. Future Perspective and Ending Notes

The progress of folate research has unfolded at an accelerated rate and new therapeutics are on the horizon [3]. The rapid development of new tools to monitor the dynamic control of metabolism through multidisciplinary approaches that incorporate synthetic biology and systems biology provide clear readouts at the transcriptional, translational, and post-translational levels. For example, one carbon metabolism was recently implicated in viral replication and infection [245]. Methionine metabolism determined viral latency by controlling the B cell EBV epigenome [246]. 

Going forward, advances that tackle challenges associated with the complexity of vitamer chemical structures and the vast number of low quantity metabolites could aid in current limitations. Between advanced metabolomics, new models of disease, isotopic labeling approaches, and sensors, new technologies will have revolutionary impacts on medicine and forge a future for dietary regimens that enhance therapeutic interventions. Over the next decade, user-friendly and affordable metabolomics-based instrumentation will play a major role in the clinical diagnosis across a spectrum of diseases and cancer to enable precision medicine. 

## Figures and Tables

**Table 1:** One carbon metabolic enzymes are regulated by post-translational modifications and cofactor availability.

Enzymes	Function	PTM	Regulation	Cofactor	Ref.
BHMT	Methionine from betaine and homocysteine	Acetylation (K232,283) Phosphorylation (T45, Y363, S366)	Homocysteine: Methionine	Zn^2+^	[18,19]
DHFR	DHF reduction to THF	Acetylation (R33, K174) Phosphorylation (S145, Y183) R-monomethylation (R29) Ubiquitylation (K47, K153)	THF	NADPH	[20,21,22,23]
MAT	SAM synthesis	R-monomethylation (R264) Phosphorylation (S114, Y296) Ubiquitylation (K351)	SAM	ATP, H_2_O, methionine	[23,24,25]
MS	Methionine synthesis	Phosphorylation (T1264)	Methionine	Cobalamin, Zn^2+^	[26]
MTHFD	Tetrahydrofolate interconversion	R-monomethylation (R37, R324, R495)	THF	ATP, NADPH, H_2_O	[27]
MTHFR	5-MTHF synthesis	Phosphorylation (S9, 10, 19, 20, 21, 23, 25, 26, 29, 30, 103, 394; T34, 94, 451; Y90)	5-MTHF	FAD	[28,29]
SHMT	5,10-MTHF and glycine synthesis	Acetylation (K271) Phosphorylation (Y34)	THF	Serine	[30]
PDXK	PLP synthesis	Acetylation (K76) Phosphorylation (S59, 164, 213, 285)	PLP	ATP	[31]
PNPO	PLP synthesis	Phosphorylation (S231, T238)	PLP	O_2_	[32,33]
TS	DHF synthesis	Phosphorylation (S114, Y153) Ubiquitylation (K169, K308)	dTMP	-	[23,34]

**Table 2:** Vitamin B9 has been implicated across a spectrum of cancers. Cellular and tissue responses are defined.

Cancer	B9	Model	Readout	Cellular and Tissue Response	Ref.
Lung	Up	Meta-analysis	Cancer incidence	MTHFR C677TT genotype correlated with increased risk MTHFR 1298CC genotype correlated with increased risk	[60]
	Down	Case-control study	Cancer incidence	MTHFR C677TT genotype correlated with decreased risk in women	[61]
Colon	Down	Meta-analysis Animal model	Folate supplement intake Serum, blood, liver, and colonic folate levels; lesion measurements; p53 methylation assessment via HpaII-PCR	Inverse correlation between supplementation and risk Deficiency enhanced carcinogenesis	[62,63,64]
	None	Case-control study Population-based study	Serum folate, B6, B12, riboflavin, and homocysteine Dietary folate, methionine, and associated B vitamin intake; Serum folate levels	No association between folate and risk No association between dietary intake of folate and risk	[65,66]
Ovarian	None	Meta-analysis	Dietary and total folate intake	No association between folate and risk	[67]
	Up	Tumor biopsy	p53 and MDM2 tissue expression	Folate receptor (FR) increases chemotherapy resistance by stabilizing MDM2	[68,69]
	Down	Meta-analysis	Dietary folate intake	Inverse association between folate and risk	[70]
Pancreatic	None	Meta-analysis	Dietary folate intake	Inconsistent results linking dietary folate intake with risk	[71]
	Down	Meta-analysis	Dietary folate intake	Decreased risk with increased dietary folate intake MTHFR 677TT associated with increased risk	[71,72,73]
Prostate	None	Meta-analysis	Serum folate levels	No association between folate and risk	[74,75]
	Up	Meta-analysis Case-control study	Serum folate levels	24% increase in risk 4% increase risk with every 5 nmol/L increase in serum folate	[76,77]
	Down	Case-control study	Serum folate, homocysteine, and B12 levels and 5,10-MTHFR polymorphism	Low folate and high homocysteine associated with increased risk	[78]
Breast	None	Meta-analysis	Dietary folate intake	No association between folate and risk	[72,79,80]
	Down/Up	Systematic review	Serum folate levels	Dietary intake between 153–400 ug/day correlated with reduced risk. More pronounced in women with high alcohol consumption	[79]

**Table 3:** Vitamin B6 has been implicated across a range of cancers. Cellular and tissue responses are defined.

Cancer	B6	Model	Readout	Cellular and Tissue Response	Ref.
AML	Up	CRISPR-Cas9 screen	Cancer incidence	AML addiction to PLP; PDXK disruption inhibited AML proliferation	[136]
	Down	Clinical	Serum PLP levels	Low vitamin B6 levels associated with increased cancer risk	[166]
Colon	Down	Population-based study HT29, LoVo, HepG2 cell lines; mouse model	Serum PLP levels Real-time PCR p21 expression; p53 protein levels	Increased cancer risk; high PLP levels decrease risk of alcohol-associated colon cancer Vitamin B6 increases p21 expression and p53 activation	[167,168,169]
	Down	Xenograft mouse model	Tumor volume	Vitamin B6 elevated in exercising mice associated with slowed tumor growth	[170]
Lung	Down	Genome-wide siRNA-based screen; A549 cell line; xenograft mouse model Case-control study	NSCLC response to anti-tumor drug cisplatin Serum PLP, folate, methionine, and cotinine levels	Vitamin B6 sensitizes cells to cisplatin; low PDXK levels correlated with poor prognosis. Low vitamin B6 levels associated with increased cancer risk	[171,172]
	Up	Case-control study Cohort study	Serum PLP, folate, and B12 levels Daily supplement doses	High vitamin B6 levels associated with increased risk High vitamin B6 levels associated with increased risk, especially in male smokers	[173,174]
	None	Systematic review	Dietary PLP intake and serum or blood PLP levels	No association between vitamin B6 and lung tumor sites	[175]
Breast	Up	Population-based case-control study	Dietary PLP intake and serum PLP levels	Breast cancer patients displayed higher serum vitamin B6 levels	[176]
	Down	Population-based case-control study	Dietary PLP intake and serum PLP levels	Vitamin B6 increase folate’s chemoprotective effect, lowering breast cancer risk	[176]
	None	Population-based case-control study	Dietary PLP intake and serum PLP levels; PCR-RFLP-based assay	No association between high vitamin B6 intake or serum levels with cancer risk	[177]
Pancreatic	Down	Meta-analysis Population-based study	Blood PLP levels Dietary PLP intake	Higher vitamin B6 levels inversely associated with cancer risk; cancer risk decreased by 9% for every 10 n/mol PLP increment Higher vitamin B6 levels inversely associated with cancer risk	[32,178]
Prostate	None	Meta-analysis Case-control study	Blood PLP levels Food-frequency questionnaire	No association between PLP and cancer risk	[175,179]
	Down	Case-control study	Dietary PLP intake	Low vitamin B6 levels associated with increased cancer risk; organ sensitivity to hormone action increased with low levels of vitamin B6	[180]
Skin	Down	B16 cell line; xenograft mouse model B16F10 cell line	Cell proliferation; tumor growth Cell proliferation	Reduction in cell proliferation when grown with 5.0 mM PN or 0.5 mM PL; tumor reduction with vitamin B6 pretreatment 500 uM PL suppressed cell growth but PN displayed a weak inhibitory effect	[181,182]
Kidney	Down	Case-cohort study	Plasma PLP levels	High vitamin B6 levels associated with decreased risk of cancer and better prognosis	[183,184]
	None	Meta-analysis	Dietary PLP intake	No association between vitamin B6 intake and kidney tumors	[175]
